# Supplementary figures and images for: Wildfire smoke knows no borders: Differential vulnerability to smoke effects on cardio-respiratory health in the San Diego-Tijuana region
Source: PLOS Glob Public Health. 2023 Jun 22;3(6):e0001886. doi: 10.1371/journal.pgph.0001886 (PMC10287006; doi:10.1371/journal.pgph.0001886)

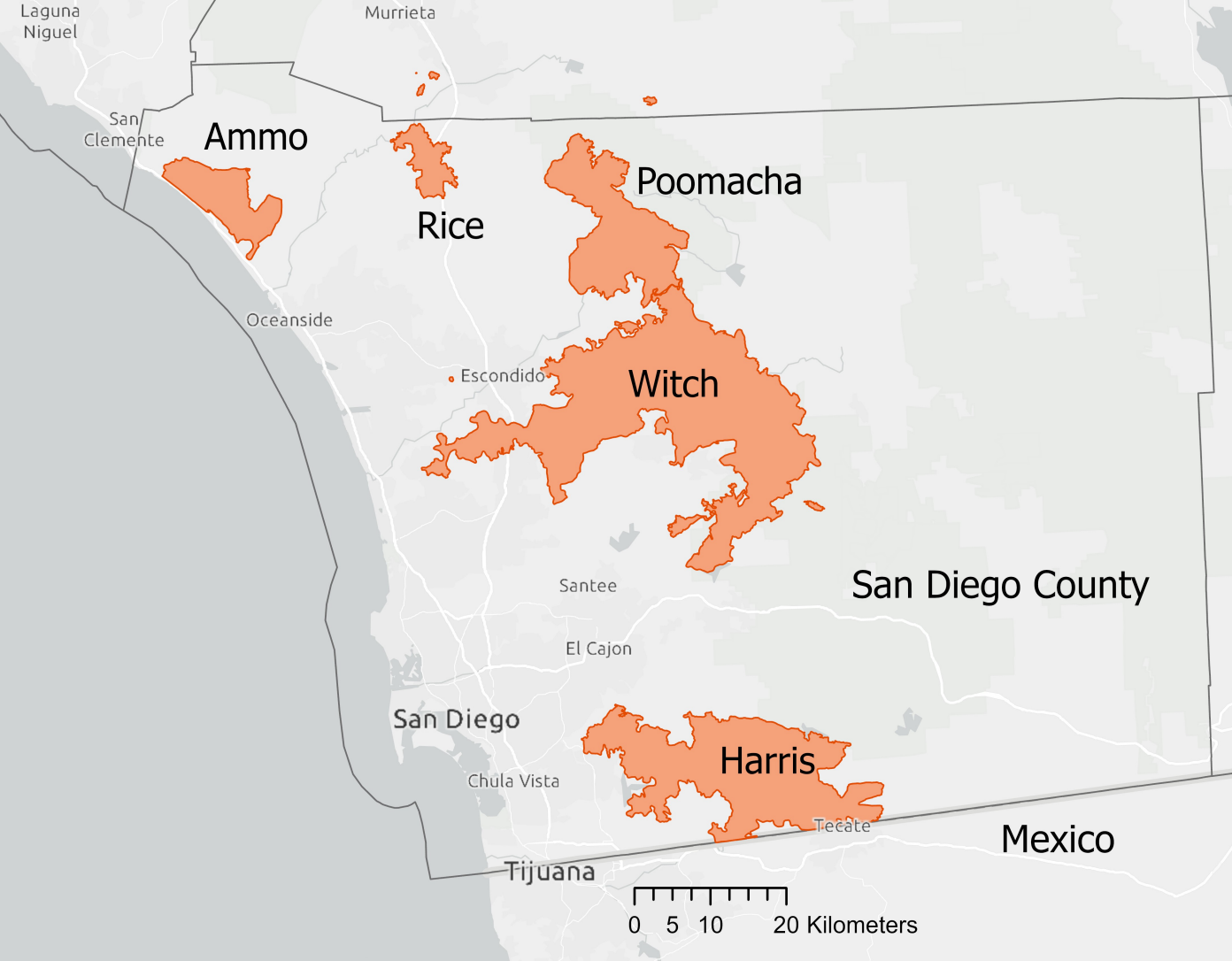

Supplement: S1 Fig — Spatial extent of Witch, Harris, Poomacha, and Rice and Ammo wildfires that burned in San Diego County, October 2007, map data from OpenStreetMap [71]. (PDF) [file pgph.0001886.s001.pdf]

— Treated — Estimated  $Y(0)$

**San Diego 20%**

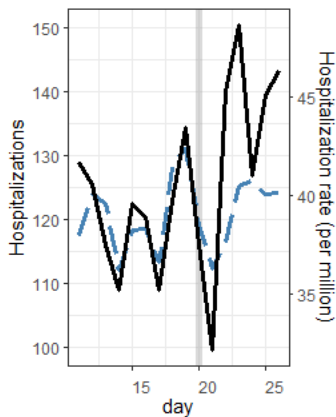

**San Diego 50%**

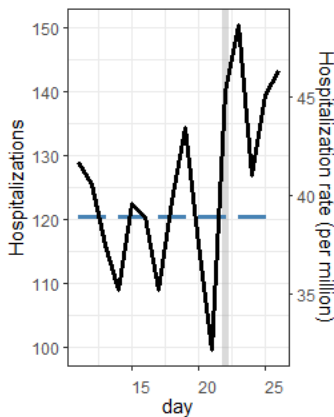

**San Diego 70%**

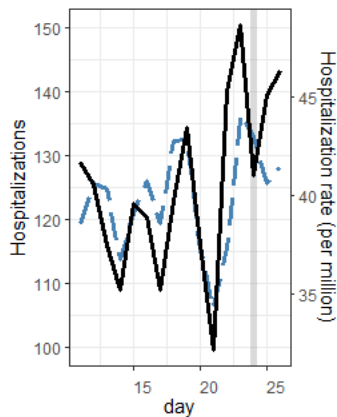

**Tijuana 20%**

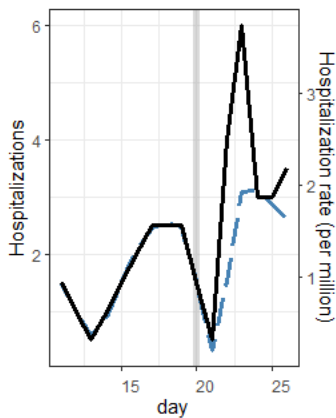

**Tijuana 50%**

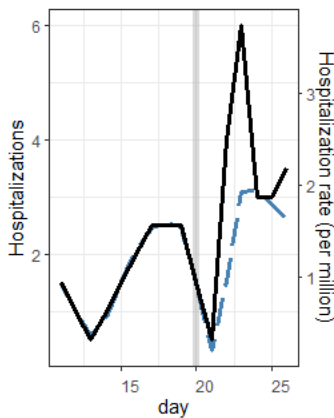

**Tijuana 70%**

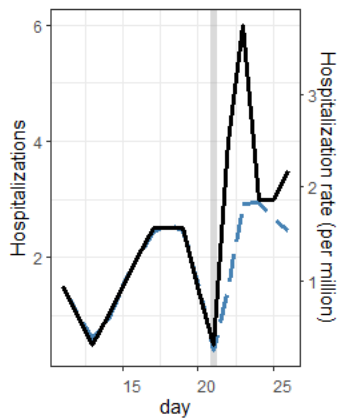

Supplement: S3 Fig — Sensitivity analyses considering 20%, 50% and 70% smoke coverage as exposure for San Diego and Tijuana. (PDF) [file pgph.0001886.s003.pdf]

### San Diego

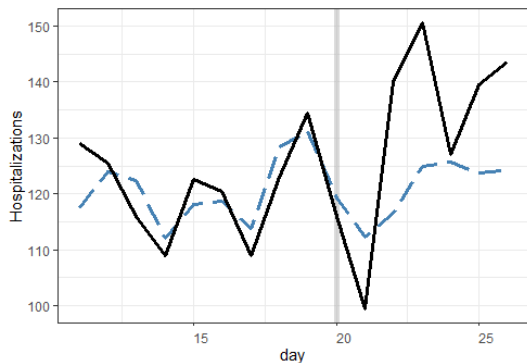

— Treated — Estimated Y(0)

### San Diego

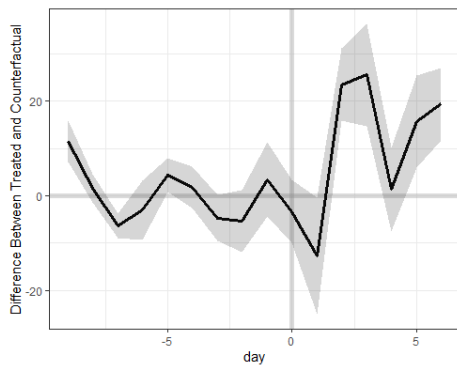

### Tijuana

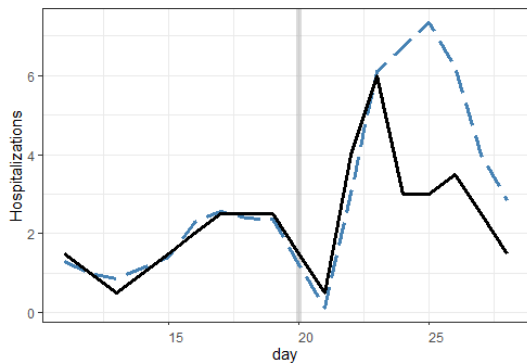

— Treated — Estimated Y(0)

### Tijuana

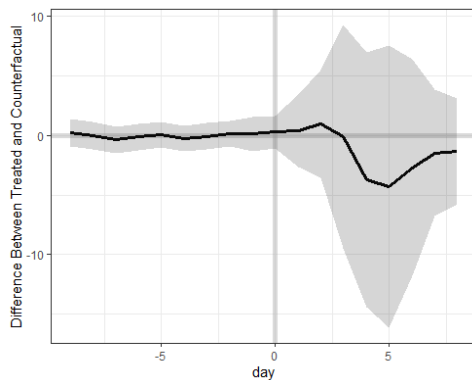

Supplement: S4 Fig — Results of sensitivity analysis including daily mean temperature as a covariate in evaluating the effect of wildfire smoke from October 2007 wildfires on cardio-respiratory hospitalizations in San Diego County and the Municipality of Tijuana. (PDF) [file pgph.0001886.s004.pdf]

# Tijuana

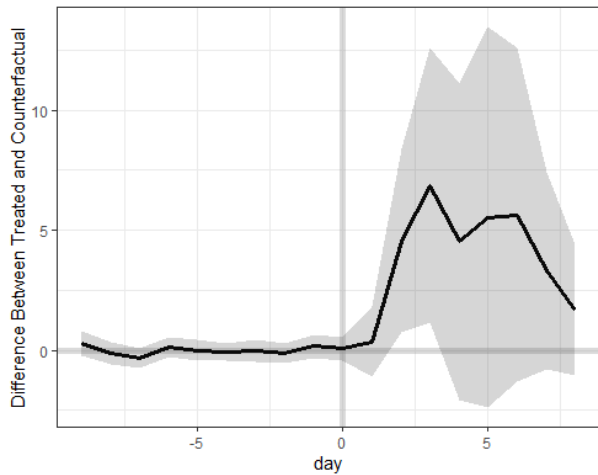

# Tijuana

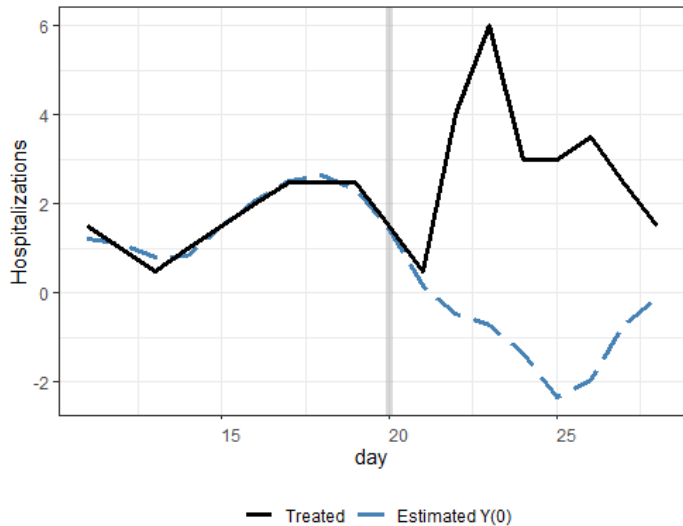

Supplement: S5 Fig — Results of sensitivity analysis including all potential controls (not excluding municipalities that had any day with 0 cases) of the effect of October 2007 wildfire smoke on cardio-respiratory hospitalization in the Municipality of Tijuana. (PDF) [file pgph.0001886.s005.pdf]
