# Supplementary material for: Wildfire smoke knows no borders: Differential vulnerability to smoke effects on cardio-respiratory health in the San Diego-Tijuana region
Source: PLOS Glob Public Health. 2023 Jun 22;3(6):e0001886. doi: 10.1371/journal.pgph.0001886 (PMC10287006; doi:10.1371/journal.pgph.0001886)
Supplement: S2 Fig — The effect of wildfire smoke on cardio-respiratory hospitalizations in San Diego and Tijuana using synthetic control methods with confidence intervals. (PDF) [file pgph.0001886.s002.pdf]

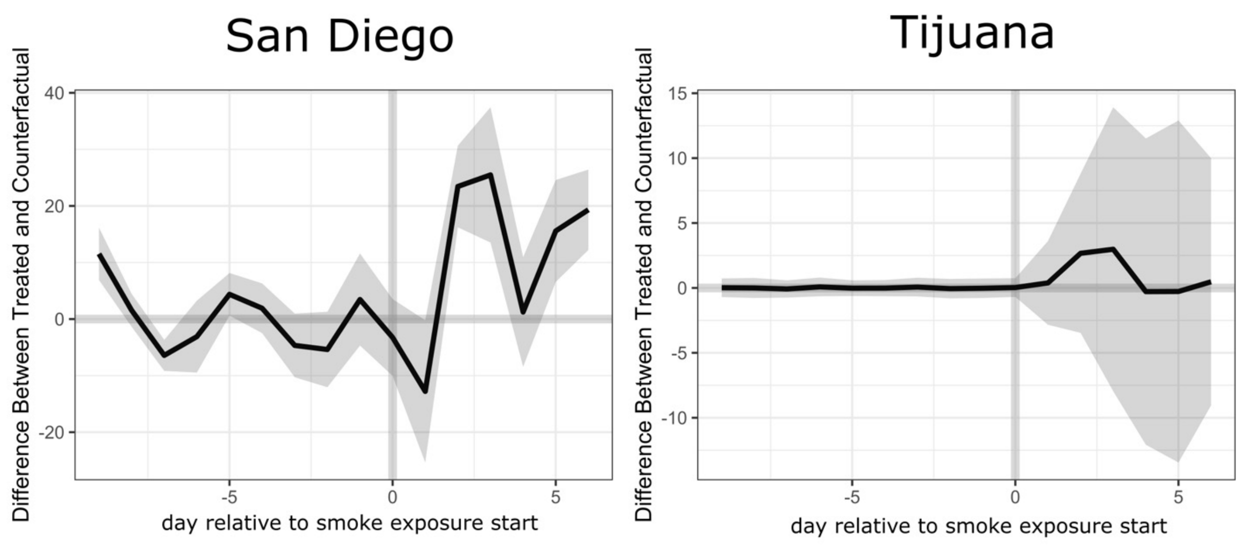
San Diego
Tijuana
Difference Between Treated and Counterfactual
40
20
0
-20
15
10
5
0
-5
-10
-5
0
5
day relative to smoke exposure start
