# Supplementary material for: Wildfire smoke knows no borders: Differential vulnerability to smoke effects on cardio-respiratory health in the San Diego-Tijuana region
Source: PLOS Glob Public Health. 2023 Jun 22;3(6):e0001886. doi: 10.1371/journal.pgph.0001886 (PMC10287006; doi:10.1371/journal.pgph.0001886)
Supplement: S1 Table — Gender and age distribution of hospitalizations in San Diego County and the Municipality of Tijuana during study period (October 11th-26th, 2007). (DOCX) [file pgph.0001886.s006.docx]

**S1 Table.** Gender and age distribution of hospitalizations in San Diego County and the Municipality of Tijuana during study period (October 11^th^-26^th^ , 2007).

|  | **San Diego** | **Tijuana** |
| --- | --- | --- |
|  | ***Count (%)*** | |
| **Age** |  |  |
| 0-14 | 132 (7%) | 10 (27%) |
| 15-64 | 631 (31%) | 18 (49%) |
| 65+ | 1,246 (62%) | 9 (24%) |
| **Sex** |  |  |
| Women | 1009 (50%) | 23 (62%) |
| Men | 1000 (50%) | 14 (38%) |
|  |  |  |
| **Total** | 2009 (100) | 37 (100) |
