# Supplementary material for: Wildfire smoke knows no borders: Differential vulnerability to smoke effects on cardio-respiratory health in the San Diego-Tijuana region
Source: PLOS Glob Public Health. 2023 Jun 22;3(6):e0001886. doi: 10.1371/journal.pgph.0001886 (PMC10287006; doi:10.1371/journal.pgph.0001886)
Supplement: S2 Table — Difference between the daily cases in each treated unit and its counterfactual for the pre-treatment period. (DOCX) [file pgph.0001886.s007.docx]

| **Difference between treated and counterfactual** | **San Diego** | **Tijuana** |
| --- | --- | --- |
| Oct 11^th^ | 11.53 | 0.016 |
| Oct 12^th^ | 1.55 | -0.002 |
| Oct 13^th^ | -6.42 | -0.079 |
| Oct 14^th^ | -3.10 | 0.078 |
| Oct 15^th^ | 4.37 | -0.013 |
| Oct 16^th^ | 1.91 | 0.066 |
| Oct 17^th^ | -4.66 | -0.054 |
| Oct 18^th^ | -5.38 | -0.024 |
| Oct 19^th^ | 3.46 | 0.030 |
| Oct 20^th^ | -3.26 | 0.386 |
