# Supplementary material for: Wildfire smoke knows no borders: Differential vulnerability to smoke effects on cardio-respiratory health in the San Diego-Tijuana region
Source: PLOS Glob Public Health. 2023 Jun 22;3(6):e0001886. doi: 10.1371/journal.pgph.0001886 (PMC10287006; doi:10.1371/journal.pgph.0001886)
Supplement: S3 Table — Controls and their weights estimated using generalized synthetic control methods. (DOCX) [file pgph.0001886.s008.docx]

| **San Diego County** | | **Municipality of Tijuana (highest weights)** | |
| --- | --- | --- | --- |
| County Number | Synthetic control weight | Municipality | Synthetic control weight |
| 103 | 0.0617 | MX09015 | 1.7048 |
| 105 | -0.0661 | MX14120 | 1.1332 |
| 11 | -0.0394 | MX09005 | 1.0402 |
| 115 | 0.0263 | MX28041 | 0.9280 |
| 15 | 0.0615 | MX30087 | 0.9025 |
| 21 | 0.0325 | MX26030 | 0.8488 |
| 3 | 0.0550 | MX11003 | 0.7813 |
| 33 | 0.0823 | MX15057 | 0.7566 |
| 35 | 0.0142 | MX15033 | 0.7565 |
| 49 | 0.0433 | MX09002 | 0.7392 |
| 51 | 0.0089 | MX30193 | 0.6637 |
| 57 | 0.0929 | MX15039 | 0.6473 |
| 63 | -0.0101 | MX08019 | 0.6295 |
| 7 | 0.4499 | MX07019 | 0.5427 |
| 89 | -0.0573 | MX11020 | 0.5178 |
| 91 | 0.0040 | MX24028 | 0.4991 |
| 93 | -0.0192 | MX23005 | 0.4702 |
|  |  | MX27004 | 0.4337 |
|  |  | MX16053 | 0.3971 |
|  |  | MX28038 | 0.3716 |
|  |  | MX01001 | 0.3617 |
|  |  | MX09011 | 0.3323 |
|  |  | MX10005 | 0.3229 |
|  |  | MX14098 | 0.3215 |
|  |  | MX28022 | 0.2896 |
|  |  | MX15104 | 0.2849 |
|  |  | MX15014 | 0.2806 |
|  |  | MX10007 | 0.2666 |
|  |  | MX15106 | 0.2553 |
|  |  | MX15058 | 0.2472 |
|  |  | MX07089 | 0.2364 |
|  |  | MX09003 | 0.2135 |
|  |  | MX14070 | 0.2029 |
|  |  | MX19039 | 0.2022 |
|  |  | MX08037 | 0.2003 |
|  |  | MX14101 | 0.1999 |
|  |  | MX07101 | 0.1758 |
