# Supplementary material for: Wildfire smoke knows no borders: Differential vulnerability to smoke effects on cardio-respiratory health in the San Diego-Tijuana region
Source: PLOS Glob Public Health. 2023 Jun 22;3(6):e0001886. doi: 10.1371/journal.pgph.0001886 (PMC10287006; doi:10.1371/journal.pgph.0001886)
Supplement: Attachment — Submitted filename: Response_to_Reviewers.docx [file pgph.0001886.s009.docx]

**PLOS Global Public Health**

**Re: Wildfire smoke knows no borders: differential vulnerability to smoke effects on cardio-respiratory health in the San Diego-Tijuana region**

Dear Editor,

We thank the reviewers for their time in efforts in providing feedback on our manuscript. Based on reviewer comments, we have added more details about the 2007 wildfires including a section for study context, clarified the methodological approach, and expanded the discussion to include additional points and limitations. We believe the manuscript has improved following these edits and we hope these changes adequately address the reviewer comments.

**Reviewer 2 comments:**

This study aimed to examine the differential effect of wildfire smoke on cardio-respiratory hospitalizations in San Diego and Tijuana using data from 2007 wildfires hospitalization. The novelty of the study lies in the use of spatial data with health data to examine this differential effect and the rigor of statistical modeling. However, I have some concerns that need clarification from the authors.

**Comment 1:** The introduction is well written, and the objectives of the paper clearly stated.

**Response:** We thank the reviewer for the positive feedback!

**Comment 2:** The current description of the October 2007 wildfires provides a good context and motivation to the study; however, I think it can be expanded. You could create a sub-section for the study context. Highlight the prevalence of wildfires in the area, their effect, studies related to the health effects of wildfires, and why the focus on the 2007 wildfires. You could also talk about the duration and the impact it had. Then narrow down to focus on the cardio-respiratory infections that may have been overlooked.

**Response:** We thank the reviewer for this comment. A study context section has been added to the methods with the following text:

*“The San Diego-Tijuana border region is between a Mediterranean and semi-arid climate (28). Most of the annual precipitation occurs in the winter, from November to March, and is subject to high variability, driving increasingly frequent droughts and floods (29). The region is affected by Santa Ana winds (SAWs), dry down-sloping winds rooted in cold air masses over the elevated Great Basin, which affect the region primarily between September and May (30). SAWs are linked to the ignition and spread of wildfires, and spreading the smoke burden during these events (32). The October 2007 wildfires was a large record-breaking event, which ignited thirteen different wildfires from Los Angeles to Tijuana; this was exacerbated by SAWs (33). This event as well as other more recent wildfires in the region highlight the vulnerability of the region to this climatic threat. The area has experienced many record-breaking wildfire events in recent years, and this is projected to continue increasing in the context of climate change.*

*Numerous studies have been conducted to understand the epidemiological effects of extreme heat and wildfire smoke on California as a whole (16, 34-36) and in San Diego specifically (17, 18, 37). Contrastingly, little to no research has been conducted in Tijuana or Baja California to understand these impacts. The proximity of these cities and the discrepancy in available evidence on this topic highlights the need to find strategies to adapt research tools and methods to each context; understanding the impacts of extreme weather events in regions with limited epidemiological data will become increasingly important.*

*In San Diego and California, it has been well established that wildfire smoke drives adverse health outcomes (16, 18, 38-42). An evaluation of the health effects of a major wildfire event that occurred in Southern California in 2003 showed increased eye and respiratory symptoms, medication use, and physician use for children in communities in Southern California (40). Recent work in Southern California has shown that PM2.5 from wildfire smoke can drive up to 10 times more respiratory hospitalizations than non-wildfire PM2.5 (16). A study specific to San Diego County found a 30% (95% CI: 26.6% to 33.4%) increase in emergency and urgent care visits at Rady’s Children's Hospital network for each 10-unit increase in PM2.5 from wildfire smoke (43). A study evaluating the economic impact of the 2007 wildfires showed that it drove medical costs by over $3.4 million (38). These are a few of the numerous studies in San Diego and California that demonstrate the health impacts of wildfire smoke and highlight the need to inform policies to limit the harmful effects of this exposure.*

*The strong health effects of wildfire smoke observed in San Diego County indicate the need to understand and reveal these impacts in Tijuana. Studying these effects in Tijuana is critical to informing early warning systems and addressing this research gap. Ideally, forecasting systems for wildfire smoke will consider environmental risk factors on both sides of the border and inform warning systems and action plans to protect populations in the entire region.”*

**Comment 3:** The authors adequately describe the two forms of data used in the analysis but there is little information on how the data were merged and the level at which these data were examined. How did the authors adjust for spatial autoregression?

**Response:** We thank the reviewer for pointing out the lack of clarity in our data and process. The data was estimated at the county (U.S.) and municipality (Mexico) level and environmental data on smoke coverage and health data on cardio-respiratory hospitalization was merged for each county/municipality. As the synthetic control methodology capitalizes on the temporality of the event (wildfire smoke occurrence) and compares the outcome (cardio-respiratory hospitalizations) in the pre-event and post-event period, each county/municipality is analyzed separately. As this is aggregated data at the county/municipality level with each being analyzed as a separate case study, there is no spatial component to this analytical approach. It would be very interesting to compare these effects across different counties/municipalities and this is an interesting avenue for future research. The following sentences have been added to the methods/discussion to clarify this:

*“The benefit of this approach is that it capitalizes on the temporality of the treatment or wildfire event to compare the pre-treatment and post-treatment hospitalization count by estimating what would have occurred if the wildfire smoke had not hit the region through the identification of synthetic controls. The trend in hospitalizations is then followed after the treatment and any difference between the treated unit and its synthetic control can be attributed to the wildfire smoke. ”*

*“Two analyses were conducted considering San Diego County and the Municipality of Tijuana separately.”*

**Comment 4:** While spatial data provide a good proxy for measuring particulate matter concentration, the authors may want to consider objectives measures of particulate matter concentrations in the area at the time. Where inaccessible, the authors may want to highlight the limitation of using only spatial data in assessing exposure to PM2.5

**Response:** We thank the reviewer for this comment and agree that using spatial coverage as the exposure metric for wildfire smoke exposure has limitations. We have added the following sentence to acknowledge this:

*“Also, smoke coverage percentages are considered a proxy for the population exposure, therefore exposure misclassification may remain.”*

**Comment 5:** It is less evident in the paper that the socio-demography of San Diego and Tijuana were considered in the computation of the models although some description of it is given in the results and discussion. From the current description, I do not see an adjusted spatial model. Kindly consider providing some information of this in the paragraph on data analysis.

**Response:** Socio-demographic characteristics of San Diego and Tijuana were not considered in the model and are added as a potential discussion for why we observe differences in our results. We have edited the discussion to clarify the hypothetic nature of this point and highlight the need to confirm this in future work:

*“Although social conditions are hypotheses for which we may see differing effects, additional research would have to consider the role of social vulnerability to confirm this as a proposed mechanism for the observed differences.”*

**Comment 6:** Please give some time to proofread the manuscript and correct all typos (there are few that need editing).

**Response:** We thank the reviewer for this comment and have carefully revised and edited the manuscript.

**Reviewer 3 comments:**

**Comment 1:** This is a very interesting article on the nexus between wildfire smoke and cardio-respiratory diseases. Authors have revealed important role of socio-demographics in modulating the effects of wildfire smoke and can be potentially useful in developing a concerted regional effort to protect populations on both sides of the border from the adverse

health effects of wildfire smoke. Findings are interesting. However, I suggest to revisit conclusion of the study and make it specific with concreate recommendations.

**Response:** We thank the reviewer for this comment. The following paragraph has been added to the discussion to expand the conclusions and recommendations:

*“To our knowledge, neither San Diego nor Tijuana has an established early warning system or action plan to protect populations during these events (64). The Air Quality Index can be used to evaluate local air and regional air quality conditions during a wildfire event, but no specific communication or intervention is activated based on these exposure levels (64). Strengthening the network of environmental health research in Baja California and San Diego can help increase the preparedness of the region in the context of climate change. Forecast systems for SAWs have been shown to be accurate for a 6-7 day lead time (65), which can be useful in the prediction of periods of high wildfire risk. Wildfire and heat wave forecasting systems could be used by policymakers to develop and implement actions to limit the morbidity and mortality attributable to extreme weather in this region. Ideally, integrated early warning systems that account for local environmental measures and predict population exposure to wildfire smoke will be implemented to protect public health on both sides of the border by taking into account local epidemiologic information.”*

**Comment 2:** I also suggest researchers to state limitations of the study in discussion section.

**Response:** We have added the following limitations to the discussion:

*“Also, smoke coverage percentages are considered a proxy for the population exposure, therefore exposure misclassification may remain.”*

*“Lastly, the hospitalization data in Tijuana is not generalizable, limiting the potential conclusions and comparisons that can be drawn from this analysis. As the hospitalization dataset from Mexico is from the public health system, it may be biased as the population served may be more susceptible. However, we felt this analysis was valuable using the available data even if it is not the ideal comparison.”*

**Reviewer 4 comments:**

This is an interesting and important study looking at cross-border health effects of wildfire smoke exposure using the indicator of hospitalizations. While the data is more limited from Tijuana and this may affect the conclusions, it is still very important to conduct these types of studies and not leave out LMIC contexts because of more limited data.

**Comment 1:** Page 7: The last line is missing a word that affects clarity (is it meant to say “is driven by” or “drives”)? “The hospitalization rate almost 30 times higher in San Diego is driven by differences the quality and generalizability of the data sources.”

**Response:** This sentence has been edited to the following:

*“The hospitalization rate almost 30 times higher in San Diego is driven by differences in the quality and generalizability of the data sources.”*

**Comment 2:** Table 1: GDP is reported in millions of dollars (so would be in $1+ trillion for San Diego and $26+ million in Tijuana) but on page 12, it states “Furthermore, the Gross Domestic Product (GDP) of San Diego County was over $1.2 million while it was just over $26,000 for the entire state of Baja California in 2010 (7).” These are very different and should be clarified which is correct (or if either are correct).

**Response:** We thank the reviewer for noticing this typo. The GDP is reported in millions of dollars, these values have been corrected in the table and in the text.

**Comment 3:** Page 13: This is a strong statement that is not supported by the methods or statistical analysis: “The socio-economic differences are likely playing an important role in increased susceptibility to wildfire smoke observed in Tijuana.” The increased hospitalizations in Tijuana, while the relative change is higher, is not statistically significant (possibly due to small sample size of hospitalizations there). In addition, the methods did not assess for a relationship between individual and neighborhood-level SES and hospitalizations or air pollution exposure, so this is really conjecture. While it is an important hypothesis and structural factors are likely influencing exposure to air pollution and health effects from this exposure, the data does not support this. In reality, the data shows that there is a statistically significant change in hospitalizations in San Diego, but not in Tijuana – there are other potential explanations for which that could be the case, including higher baseline co-morbidities in San Diego population (this is a hypothesis/example).

**Response:** We thank the reviewer for this comment. We agree that these statements are purely hypotheses for the observed differences, which would have to be confirmed in future research. We have edited the discussion to be more nuanced in our conclusions and highlight the need for further work to confirm these potential relationships including adding the following sentence:

*“Although social conditions are hypotheses for which we may see differing effects, additional research would have to consider the role of social vulnerability to confirm this as a proposed mechanism for the observed differences.”*

**Comment 4:** Page 13: “Warning systems to forecast wildfire smoke exposure have potential to decreasing the burden; smoke-related public health messaging to stay indoors can prioritize at-risk populations (45).” I think this is supposed to say, “have potential to decrease”

**Response:** We thank the reviewer for pointing out this typo, it has been corrected in text.

**Comment 5:** Pg 14-15: Would add to limitations the much smaller number of hospitalizations in Tijuana and how this many have impacted the data. In particular, there may be skewed data there given that hospitalizations were only noted in public hospitals, which presumably may serve lower-income and more vulnerable patients. This could exaggerate the finding of relative increased hospitalizations during the study period in this population.

**Response:** We agree that it is important to highlight this limitation. The following sentences have been added to the discussion:

*“Lastly, the hospitalization data in Tijuana is not generalizable, limiting the potential conclusions and comparisons that can be drawn from this analysis. As the hospitalization dataset from Mexico is from the public health system, it may be biased as the population served may be more susceptible. However, we felt this analysis was valuable using the available data even if it is not the ideal comparison.”*
